# Reduction of Hysteresis in Hybrid Perovskite Transistors by Solvent-Controlled Growth

**DOI:** 10.3390/ma14102573

**Published:** 2021-05-15

**Authors:** Farjana Haque, Ravindra Naik Bukke, Mallory Mativenga

**Affiliations:** Department of Information Display, College of Sciences, Kyung Hee University, Seoul 02447, Korea; farjana@khu.ac.kr (F.H.); bukke@tft.khu.ac.kr (R.N.B.)

**Keywords:** hybrid perovskite, solvent-controlled growth, hysteresis

## Abstract

The effect of crystallization process speed on the morphology of solution-processed methyl ammonium lead iodide (MAPbI_3_) thin films is investigated. Crystallization speed is controlled by varying the number of annealing steps, temperature, and resting time between steps. The resting period allows solvent-controlled growth (SCG) in which crystallization progresses slowly via an intermediate phase—during which solvents slowly evaporate away from the films. SCG results in fewer residues, fewer pinholes, and larger grain sizes. Consequently, thin-film transistors with SCG MAPbI_3_ exhibit smaller hysteresis in their current-voltage characteristics than those without, demonstrating the benefits of SCG toward hysteresis-free perovskite devices.

## 1. Introduction

Organic–inorganic hybrid perovskites such as methylammonium lead iodide (CH_3_NH_3_PbI_3_ or MAPbI_3_) have recently emerged as promising candidates for high-performance and low-cost optoelectronic devices [1,2]. In solar cells, these materials have excelled as light harvesters, reaching record power conversion efficiencies of over 20% in their early stages of development [3]. Such tremendous potential is mainly due to their unique properties, which include tunable bandgaps, high photoluminescence quantum yields, high absorption coefficients, and low density of electronic states, despite low-temperature (<150 °C) processing [2,4,5,6,7]. Besides photovoltaics, organic–inorganic hybrid perovskites are also appealing for other optoelectronic devices, and research on photodetectors [8], thin-film transistors (TFTs) [9], and light-emitting diodes (LEDs) [10] has already made rapid progress.

However, instability in the performance of these devices, especially the hysteresis in their current-voltage (I-V) characteristics, still limits their application in commercial products [8,9,10,11,12,13,14,15,16,17,18,19]. This hysteresis has been linked to ferroelectricity or defect-mediated drift-diffusion of ions in the perovskite thin films during device operation [15,16,17,18,19]. In field-effect devices such as TFTs, the migration of these ions results in a partial screening of the applied gate field, yielding reduced gate modulation of electronic charges [17,18,19,20,21]. Similarly, ion migration also leads to a significant screening of the applied electric field in solar cells and LEDs, leading to measurement speed, time, or history-dependent hysteresis in their I-V characteristics [13,16,18].

The morphology of the perovskite thin films plays a crucial role in the stability of the devices. In particular, the defect-mediated drift-diffusion of ions occurs via grain boundary defects [22,23]. As the perovskite thin films are formed by low temperature solution processes, the formation of polycrystalline films with grain boundaries is unavoidable [22,23]. Structural disorder at the grain boundaries results in defect states such as dangling bonds, elemental vacancies, voids, and pinholes. Besides hysteresis, these defects also have the potential to degrade carrier transport properties of the perovskite thin films, especially the carrier mobility.

Many researchers have attempted to eliminate or counteract the hysteresis via solvent engineering [24,25,26], insertion of self-assembled monolayers (SAMs) [16], suppression of interfacial ferroelectricity [18], doping [19], defect passivation [20], additives [24,25,26,27,28,29], ambient air processing [26,27,28], and so forth [30,31,32]. However, despite all these efforts, the current situation is still far from the complete annihilation of the hysteresis problem.

In this study, we investigate the effects of crystallization process speed on the morphology of the hybrid perovskite methyl ammonium lead iodide (MAPbI_3_) and its impact on hysteresis in the electrical characteristics of ambient-air-processed MAPbI_3_ thin-film transistors (TFTs). As crystallization speed can be varied by controlling the speed at which solvents evaporate after spin coating, we vary the annealing temperature, the number of annealing steps, and the interval between steps to achieve various crystallization speeds. We show that adding resting intervals between steps enables solvent-controlled growth (SCG) where crystallization progresses via an intermediate phase during which solvents slowly evaporate away from the films. As a result, SCG decreases the total number of defects and grain boundaries in MAPbI_3_—thereby significantly reducing hysteresis in MAPbI_3_ TFTs. This work demonstrates a simple strategy to obtain hysteresis-free perovskite devices in ambient air.

## 2. Materials and Methods

Using the Laurell WS-400B spin coater (Laurell, North Wales, UK), we spin-coated several MAPbI_3_ films on bare glass substrates at room temperature in ambient air (relative humidity of 50 ± 5% and temperature of 25 ± 2 °C). For process simplicity and for better ambient air stability, the MAPbI_3_ films were deposited in ambient air as described previously in [24]. We obtained the precursor solution by dissolving powdered MAI (99.999%, Greatcell Solar, Queanbeyan, Australia) and PbI_2_ (99%, Merck Korea Ltd./Sigma-Aldrich Korea Ltd., Incheon, Korea) (MAI:PbI_2_ = 1:1) in a mixed solvent composed of gamma-butyrolactone (GBL) (99%, Merck Korea Ltd./Sigma-Aldrich Korea Ltd., Incheon, Korea) and dimethyl sulfoxide (DMSO) (99.9%, Merck Korea Ltd./Sigma-Aldrich Korea Ltd., Incheon, Korea), followed by stirring for 12 h at 100 °C in ambient air. Spin-coating speed and time were 6000 rotations per minute (rpm) and 70 s, respectively. These conditions yielded approximately 200-nm-thick MAPbI_3_ films. 

To control the crystallization speed, we annealed the films on a hot plate in ambient air at temperatures ranging from 70 to 160 °C. We also varied the number of annealing steps from 1 to 5 and analyzed film morphology by X-ray diffraction (XRD) (Malvern Panalytical Ltd., X’Pert PRO, Royston, UK), X-ray photoelectron spectroscopy (XPS) (ULVAC-PHI, INCORPORATED, PHI 5000 VersaProbe, Kanagawa, Japan), scanning electron microscopy (SEM) (Hitachi High Technologies, S-4700, Tokyo, Japan), and atomic force microscopy (AFM) (Park Systems, XE-100, Suwon, Korea). To minimize the influence of the temperature ramping in the plate heating, we pre-heated the hot plate to the desired temperature before placing the samples on it. 

**Figure 1:**
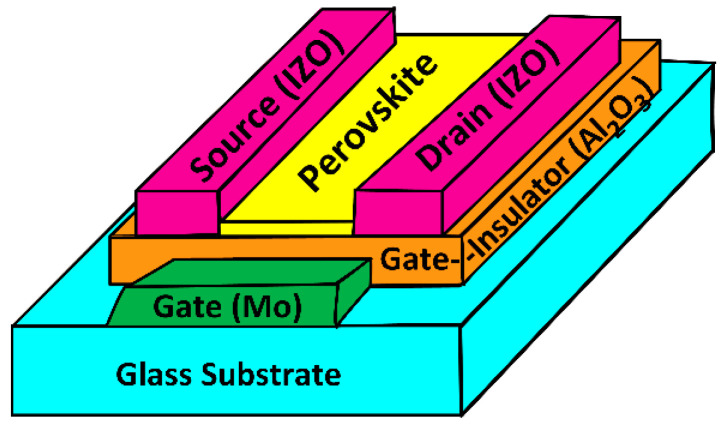
Schematic structure of MAPbI_3_ TFTs.

To investigate the hysteresis in I-V characteristics, we fabricated MAPbI_3_ TFTs with the inverted coplanar structure (Figure 1). The gate electrode was a 40-nm-thick Mo layer deposited by sputtering at 280 °C. The TFTs employed 40-nm Al_2_O_3_ thin films as a gate insulator (G.I.) and 60-nm indium zinc oxides (IZO) (280 °C by sputtering) as source (S) and drain (D) electrodes. The capacitance of the Al_2_O_3_ layer per unit area (C_ox_) was estimated to be approximately 200 nF/cm^2^. We prepared the Al_2_O_3_ solution by mixing aluminum chloride (AlCl_3_) (Merck Korea Ltd./Sigma-Aldrich Korea Ltd. Incheon, Korea), acetonitrile (35%) (Merck Korea Ltd./Sigma-Aldrich Korea Ltd. Incheon, Korea), and ethyleneglycol (65%) (0.2 M) (Merck Korea Ltd./Sigma-Aldrich Korea Ltd. Incheon, Korea). The solution was spin-coated at 2000 rpm for 30 s followed by curing at 250 °C for five minutes (performed to evaporate the solvent) and 5 min UV (NOVASCAN PSD-series, from Novascan Tech Inc., Boone, NC, USA) ozone treatment (wavelengths of 185 and 254 nm, generated by a mercury vapor lamp). During treatment, the 185 nm UV light decomposed atmospheric oxygen molecules (O_2_) to oxygen-free radicals (·O), which effectively performed oxidative treatments on the surface of the Al_2_O_3_. The O can react with O_2_ to form ozone (O_3_), which decomposes back to ·O and O_2_ due to the presence of the 254 nm UV light [33,34,35]. These reactive ·O radicals are highly effective at removing organic residuals from metal oxide film surfaces, increasing the metal-oxygen bonds, and reducing oxygen-related defects. UV treatment thus results in a smooth surface morphology with less defects [33,34,35].

For film densification, we performed a final anneal for 2 h at 350 °C. We defined the source and drain electrodes by patterning a 60-nm-thick IZO layer deposited at 280 °C via sputtering. To avoid pattering, we spin-coated a positive photoresist (PR) (Sigma-Aldrich, Inc., Steinheim, Germany) layer on top of the source and drain electrodes and patterned it to form “banks” into which we spin-coated the MAPbI_3_. Several TFTs samples were fabricated using MAPbI_3_ films grown under different conditions.

We retrieved field-effect mobility (µ_FE_) and subthreshold voltage, respectively, from the slope and intercept of the linear regression line of the |I_D_|^1/2^ (V_DS_) plot in the saturation regime (V_DS_ > V_GS_-V_TH_). V_GS_, V_DS_, and I_D_, denote the gate voltage, drain voltage, and drain current, respectively. We took the subthreshold swing (SS) as the minimum of (d log (I_D_)/d V_GS_)^−1^ and the hysteresis voltage (V_HYS_) as the difference between V_GS_ values corresponding to I_D_ equal to 1 nA in the forward and reverse sweeps.

## 3. Results and Discussion

### 3.1. Film Morphology

**Figure 2:**
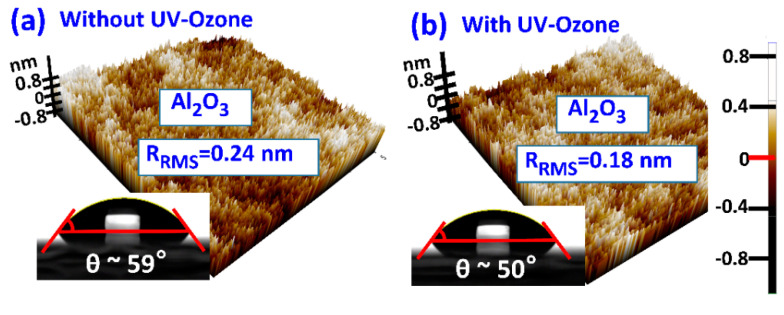
Atomic force microscopy (AFM) images and contact angles of Al_2_O_3_ films; (**a**) before and (**b**) after UV-ozone treatment.

For better adhesion, we treated the surface of the Al_2_O_3_ with UV-ozone for 10 min before MAPbI_3_ deposition (Figure 2). Surface properties of Al_2_O_3_ films with and without UV-ozone treatment were analyzed using atomic force microscopy (AFM) and contact angle (CA) measurements. Removal of organic solvent residues by the UV-ozone treatment decreases surface roughness as root mean square roughness (R_RMS_) decreased from 0.24 nm to 0.18 nm. This also improves the wettability of the Al_2_O_3_ film. 

The contact angle of the Al_2_O_3_ G.I. decreased from 59° to 50° after UV-ozone treatment (AHTECH LTS., Gyeonggi-do, Korea), indicating higher surface energy (Figure 2). We calculated the surface energy (γ_s_) of the Al_2_O_3_ film before and after UV-ozone treatment using the equation γ_s_ = (γ_w_/4) × (1 + cosθ)^2^ where θ is the contact angle at equilibrium and γ_w_ is the water surface free energy and obtained the values 23.96 and 30.60 mJ/m^2^, respectively. Higher surface energy indicates better interfacial qualities between the MAPbI_3_ semiconductor and the Al_2_O_3_ G.I. It is important to note that without UV-ozone treatment the low wettability of the substrate results in fewer nucleation sites. Although fewer nucleation sites can lead to slightly larger grain sizes, the films without UV-ozone treatment cannot achieve functioning TFTs because they contain a large number of voids and pinholes, which deter lateral conduction in the thin films [36].

**Figure 3:**
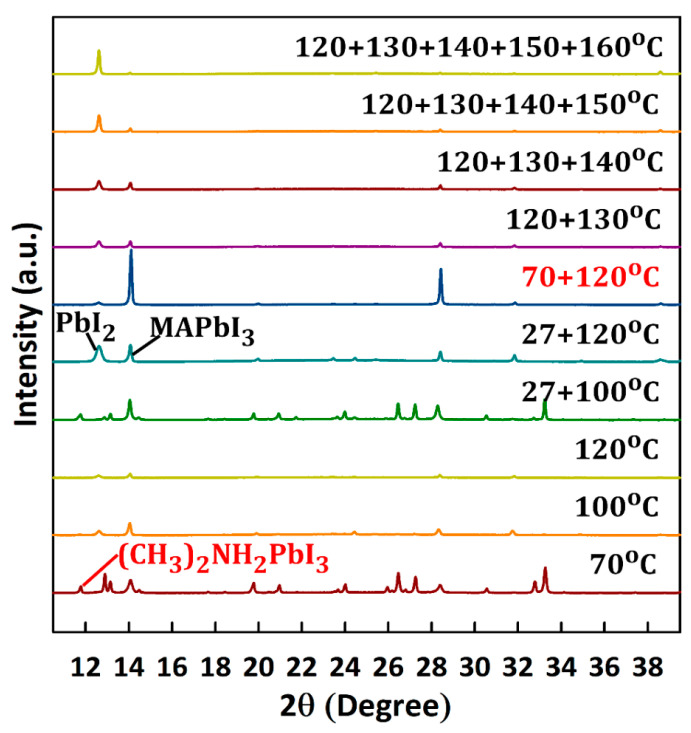
X-ray diffraction (XRD) patterns of MAPbI_3_ films grown via one-step and multistep annealing processes at various temperatures. A “+” sign indicates a multistep process in the order of appearance. For instance, 70 + 120 °C stands for a two-step annealing process involving an initial annealing at 70 °C followed by second annealing at 120 °C. Each annealing step lasted for 10 min and the multistep annealing processes involved a 1-h resting time (at room temperature in a vacuum) between steps.

Figure 3 shows the XRD patterns of MAPbI_3_ films fabricated via one-step and multistep annealing processes. A “+” sign indicates a multistep process in the order of appearance. For instance, 70 + 120 °C stands for a two-step annealing process involving an initial annealing at 70 °C followed by second annealing at 120 °C. Each annealing step lasted for 10 min and the multistep annealing processes involved a 1-h resting time (at room temperature in a vacuum) between steps. 

**Figure 4:**
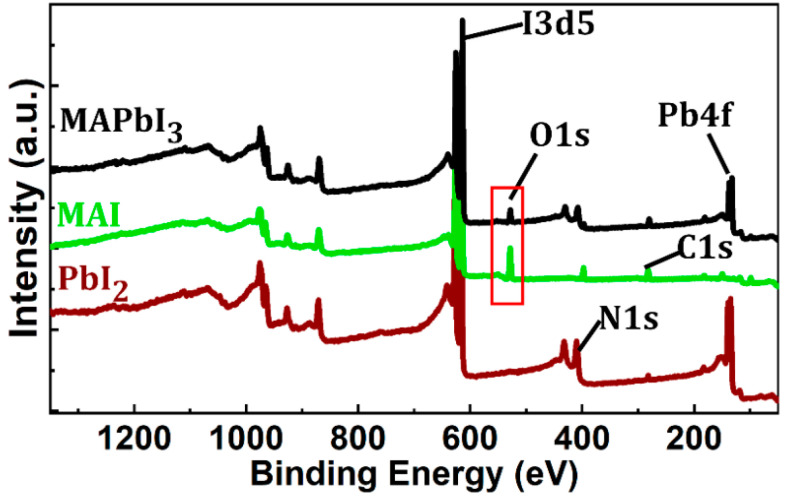
X-ray photoelectron spectroscopy (XPS) results for the one-step 120 °C annealed film.

Compared to that of films annealed at 70, 100, or 120 °C, the ratio of the MAPbI_3_ peak-to-PbI_2_ peak of the film successively annealed at 70 and 120 °C (70 + 120 °C) was the highest, indicating the superiority of multistep to one-step annealing processes. The MAPbI_3_ peak-to-PbI_2_ peak ratio dramatically decreased at temperatures >120 °C for both one-step and multistep annealing processes. It is important to note that multistep processes with a maximum temperature of ≤120 °C (e.g., 70 + 100 + 120 °C) yielded similar results to the 70 + 120 °C process. Additionally, resting time ≥ 1 h resulted in similar results (not shown here). As we deposited the MAPbI_3_ in ambient air, oxygen in the air also strongly influenced the crystal quality. Previous studies have shown the presence of oxygen during annealing to reduce defects without affecting the surface morphology [37,38]. Moreover, it is suggested that oxygen reduces grain boundary defects, yielding better grain-to-grain connection, which is essential for good lateral conduction [24]. The presence of oxygen in the films (Figure 4) was consistent with defect passivation during ambient air processing [21,24,25,38,39] and evaporation of MA via oxygen bonding at temperatures greater than 120 °C (Figure 3).

The 70 + 120 °C annealing process was superior due to the existence of solvent-controlled growth (SCG), which occurred during the resting period. For SCG to occur, crystal seeds are essential, and their formation requires thermal energy [40]. Experimentally, we found temperatures around 70 °C to be sufficient for the seed formation. Given the high boiling points of GBL and DMSO (204 and 189 °C, respectively) [24], high-temperature annealing is required after SCG to effectively complete crystallization. This temperature should be around 120 °C, according to Figure 3. As multistep processes with a maximum temperature of ≤120 °C yielded similar results, we chose the two-step annealing process (70 + 120 °C) for TFT fabrication to keep the process simple.

**Figure 5:**
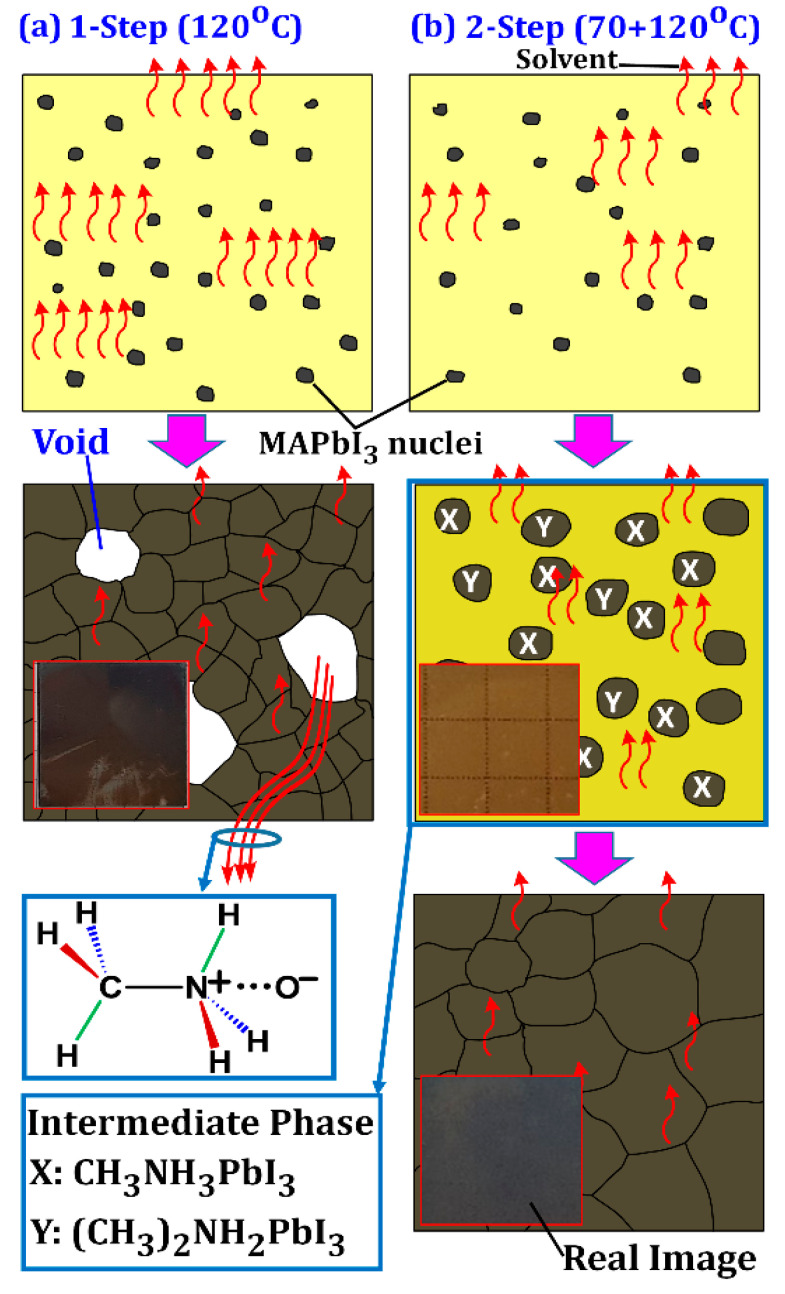
Mechanism of crystal formation in MAPbI_3_ by (**a**) one-step and (**b**) two-step annealing processes.

Figure 5a,b depicts crystallization process mechanisms during one-step and two-step annealing processes, which respectively represent fast and slow crystallization. In the case of the one-step annealing at 120 °C the color of the thin films turned blackish immediately after placing them on the hotplate (see real image in Figure 5a). This indicates rapid growth of MAPbI_3_ poly crystals with a bandgap of ≅1.72 eV. As air bubbles can be easily trapped during processing in ambient air, rapid solvent evaporation may result in pinhole formation. Additionally, excessive thermal energy may result in MA evaporating with the solvents or via oxygen bonding (Figure 4), leading to void formation in the film (Figure 5a). Extension of the annealing time or additional annealing steps at higher temperatures only results in additional evaporation of MA, leaving behind PbI_2_. MA is prone to evaporation due to its low boiling point and low sticking properties. 

In the case of the two-step annealing process, crystallization progresses slowly via an intermediate state (Figure 5b). The XRD pattern of the 70 °C film shows that this intermediate phase was composed of both MAPbI_3_ (CH_3_NH_3_PbI_3_) and methyl ammine lead iodide (CH_3_)_2_NH_2_PbI_3_ crystals (Figure 3). During the intermediate phase, the film was very transparent, so much so that the cutting board under it was clearly visible (grid lines in real image), which further suggests the incomplete formation of MAPbI_3_. When the samples were left to rest for the duration of 1 h or longer after the 70 °C annealing, crystallization progressed slowly as solvents slowly evaporated from the film. Upon annealing at 120 °C after the resting period, (CH_3_)_2_NH_2_PbI_3_ disappeared and only the MAPbI_3_ peak appeared strong, indicating complete crystallization (Figure 3, 70 + 120 °C).

**Figure 6:**
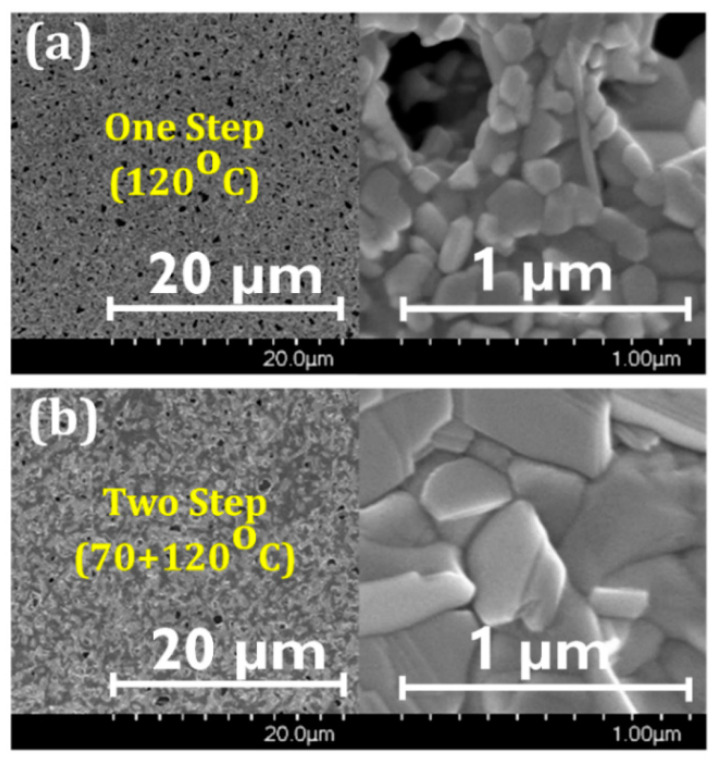
Scanning electron microscopy (SEM) images of MAPbI_3_ films fabricated with (**a**) one-step and (**b**) two-step annealing processes.

SEM images of the one-step and two-step annealed films confirmed this mechanism. The film annealed at 120 °C contained many voids and/or pinholes as a consequence of the rapid growth (Figure 6a). Fewer voids existed in the 70 + 120 °C film (Figure 6b) as most of the solvents slowly evaporated during the resting period, thereby minimizing the rapid loss of precursors during the second 120 °C annealing step. Furthermore, rapid crystallization from numerous crystal seeds led to smaller grain size in the 120 °C film than 70 + 120 °C film. If the films are left to rest after spin-coating without providing sufficient heat energy through annealing at 70 °C, the intermediate phase will not occur (compare 120 °C and 27 + 120 °C films in Figure 3). Therefore, the first step of annealing at 70 °C is necessary to provide sufficient energy for the crystal seed formation.

Similar results were observed for the inorganic perovskite CsPbI_3_ where a δ-phase CsPbI_3_ was observed upon resting the precursor films in a nitrogen-filled glovebox for several tens of minutes [41]. The formation of this additional phase resulted in films becoming more continuous and having a reduced number of pinholes and larger gain size. Additionally, solar cells employing the CsPbI_3_ films obtained by SCG displayed higher efficiency and could tolerate above 500 h of continuous light soaking [41].

In addition to the crystallization speed, thermal stress also plays a role in the morphology of perovskite films. Perovskite films can accumulate residual stress during processing. Initially, the precursor solution moves freely without any constraint during the spin-coating process due to its low viscosity and lack of interaction with the substrate. However, when the solvent evaporates during the annealing process, conversion into the crystalline perovskite structure enhances chemical interactions with the substrate. The film is thus constrained from expanding or contracting freely, leading to film stress. This constraint is large when the thermal expansion coefficient (CTE) of the film is larger than that of the substrate [42]. This is the case for halide perovskites that have a CTE (approximately 50 × 10^−6^ K^−1^) that is five times larger than that of glass (approximately 10 × 10^−6^ K^−1^) [43,44]. When the samples were cooled to room temperature after annealing, the perovskite film was constrained from contraction by the underlying glass substrate, resulting in tensile stress building up in the perovskite film. Note that the layer immediately below the perovskite was a 40-nm-thick Al_2_O_3_ layer, which was much thinner than the glass (approximately 0.6 mm). The stress was thus determined largely by the glass.

Rolston et al. found the residual stress to have a linear relationship with the temperature at which the film is annealed with higher annealing temperatures resulting in larger stress values [42]. They attributed the correlation between the residual stress and annealing temperature largely to the CTE mismatch between the film and substrate and calculated the predicted stress, σ_ΔT_, by

(1) $$\sigma_{\Delta T}=\frac{E_{p}}{1-v_{p}}\left(\alpha_{s}-\alpha_{p}\right)\Delta$$

where E*_p_* is the perovskite modulus, ν_p_ is Poisson’s ratio of the perovskite, α*_s_* and α*_p_* are the respective thermal expansions of the substrate and perovskite, and ΔT is the temperature gradient when cooling. Using an E*_p_* of approximately 12.5 GPa and ν*_p_* = 0.3 [42], σ_ΔT_ is approximately 31 and 65 MPa, respectively, for annealing at 70 and 120 °C. Therefore, the one-step annealing process (120 °C) resulted in initial residual film stress that was much larger than that of the two-step annealing process (70 + 120 °C). Although the two-step annealed sample also underwent annealing at 120 °C, the stress was expected to be less due to the presence of the intermediate phase. Residual film stress can have a significant effect on the optoelectronic properties of the perovskite films and contribute to heat, light, and moisture-based chemical degradation as well as fractures in the device [45,46].

### 3.2. TFT Current-Voltage Characteristics

**Figure 7:**
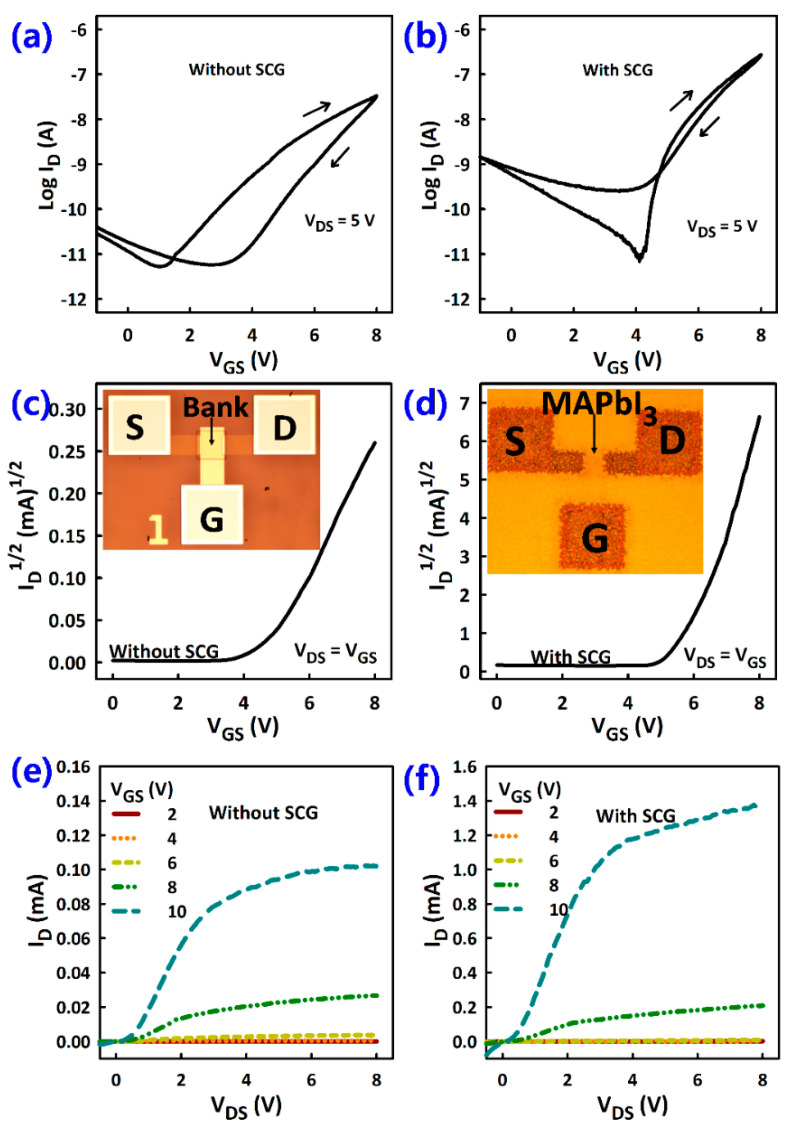
Typical current-voltage (I-V) characteristics of MAPbI_3_ TFTs with one-step (without SCG) and two-step (with SCG) annealed MAPbI_3_ films. (**a**,**b**) Transfer characteristics with forward and reverse sweeps. (**c**,**d**) Diode characteristics measured with shorted gate and drain electrodes (i.e., saturation regime). (**e**,**f**) Output characteristics. The TFTs had channel width of 100 µm and channel length of 20 µm. Insets of c and d are optical images of a MAPbI_3_ TFT before and after spin-coating MAPbI_3_, respectively.

Transfer, diode (saturation), and output characteristics of the TFTs were measured as shown in Figure 7. It is interesting to see that hysteresis in the transfer characteristics was smaller in TFTs with SCG MAPbI_3_ than those without (Figure 7a,b). Hysteresis is due to vacancy-mediated migration of ions in the MAPbI_3_ [15,16,17]. Given that most of these vacancies occur in grain boundaries or around voids, reduction of hysteresis is consistent with the larger grain size and fewer voids in the SCG-based MAPbI_3_. 

Hysteresis can also originate from ferroelectric effects, trapping at the G.I./MAPbI_3_ interface, or ion migration inside the G.I. Given that the two sets of TFTs were fabricated with an identical G.I. process, the difference in their hysteresis characteristics is mainly due to changes in the MAPbI_3_ and not the aforementioned. I^−^, CH_3_NH_3_^+^, and Pb_2_^+^ are the three ions in MAPbI_3_, and the activation energy for vacancy-mediated migration is the lowest for I^−^ (0.1–0.6, 0.46–0.84, and >2 eV, respectively) [47,48,49]. I^−^ was thus believed to dominate ion drift-diffusion in MAPbI_3_. Reduced hysteresis in SCG MAPbI_3_ TFTs was thus consistent with reduced drift-diffusion of I^−^ due to the effective reduction in the total number of defects and grain boundaries. These results were consistent with a previous report on lead-free layered perovskite transistors where grain boundary passivation and grain size enlargement were found to reduce hysteresis and increase TFT reproducibility and reliability [50].

**Figure 8:**
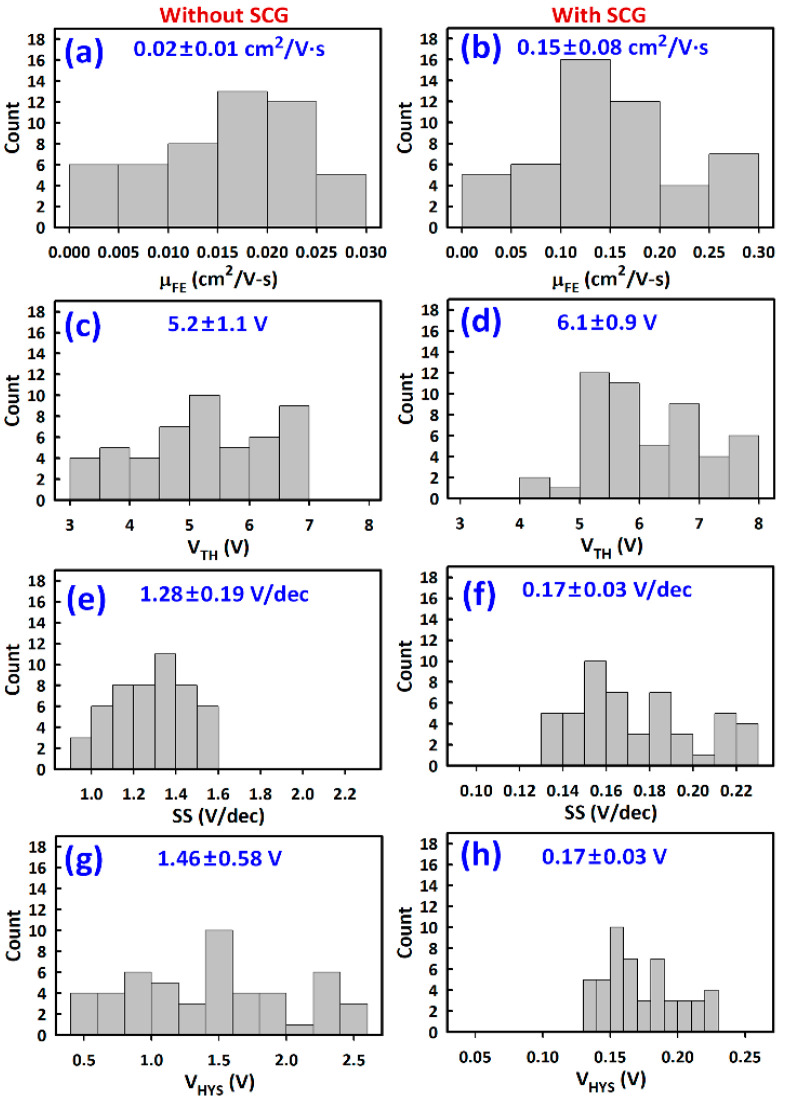
TFT Parameters extracted from 100 devices with and without solvent-controlled growth (SCG). (**a**,**b**) Field-effect mobility (µ_FE_). (**c**,**d**) Threshold voltage (V_TH_). (**e**,**f**) Subthreshold swing (SS). (**g**,**h**) Hysteresis voltage (V_HYS_). The first and second numbers inside the figures (A ± B) are the mean and standard deviation, respectively.

The diode characteristics measured by shorting the gate and drain electrodes showed a steeper slope for TFTs with SCG than those without (Figure 7c,d). The steeper slope is an indication of higher µ_FE_ as shown in Figure 8. The output characteristics also showed about an order of magnitude higher current in TFTs with SCG than those without, consistent with better film morphology for TFTs with SCG (Figure 7e,f). The current crowding at low V_DS_ values was also more pronounced in the TFTs without SCG than those with SCG, indicating higher resistance at source/drain contacts of the former—possibly due to the presence of many voids.

Owing to the good film morphology achieved by SCG, the µ_FE_ improved from 0.02 ± 0.01 to 0.15 ± 0.08 cm^2^/V·s (Figure 8a,b). The V_TH_ values were 6.1 ± 0.9 and 5.2 ± 1.1 V, respectively, for TFTs with and without SCG (Figure 8c,d). The slightly lower V_TH_ in TFTs without SCG could be related to a slightly different surface potential due to defects or mobile ions. There were evidently more defect states in the TFTs without SCG than those with SCG, given that SS of the former (1.28 ± 0.19 V/dec) was approximately ten times larger than that of the latter (0.17 ± 0.03 V/dec) (Figure 8e,f). The total trap density (N_T_) can be calculated from N_T_ = {SS(log(e))/(k_B_T/q) − 1}(C_ox_/q) where k_B_, T, and q are respectively the elementary electric charge, Boltzmann’s constant, and temperature in kelvins [51]. N_T_ values of the TFTs with and without SCG were approximately 1.29 × 10^12^ and 1.06 × 10^13^ cm^−^^2^·V^−1^, respectively [51,52]. The difference in N_T_ was mainly due to the bulk states, given that the TFTs employ the same gate insulator. 

**Figure 9:**
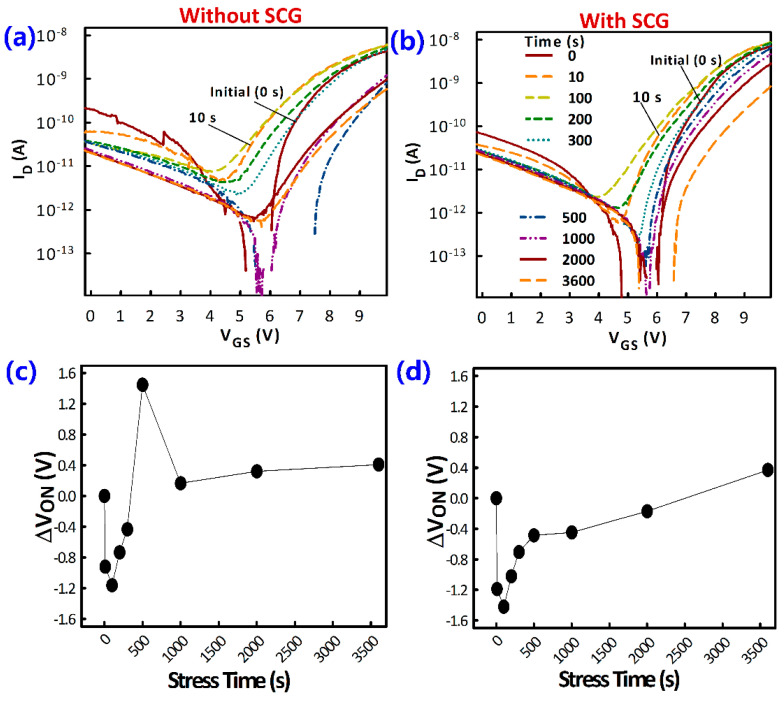
Positive bias stress (PBS) stability of MAPbI_3_ TFTs. (**a**,**b**) Transfer characteristics of the TFTs at various stages of the stress period. (**c**,**d**) Turn-on voltage shift (ΔV_ON_) as a function of stress time. The V_ON_ is taken as the V_GS_ at which the I_D_ starts to monotonically increase.

Positive gate bias stress (PBS) stability of the MAPbI_3_ TFTs was also investigated. The PBS was achieved by applying a constant V_GS_ of 10 V to the TFTs while grounding the source and drain electrodes for 3600 s. The PBS was interrupted at different points to measure the transfer characteristics as shown in Figure 9.

Consistent with having fewer defects, the TFTs with SCG showed better PBS stability compared to those without. PBS results in trapping of carriers at the semiconductor/gate-insulator interface. These trapped carriers screened the gate field, resulting in the reduction of the effective applied V_GS_, which led to positive the turn-on voltage shift (ΔV_ON_). However, the shifting of the V_ON_ of the MAPbI_3_ TFTs investigated herein was bidirectional, consistent with the contribution of other forms of hysteresis, particularly due to ion migration. The fluctuation of the V_TH_, especially at the early stages of the PBS, was much larger for the TFT without SCG—underscoring the importance of defect reduction by SCG. The similar ΔV_ON_ after 3600 s can be explained by the fact that the TFTs employed the same gate insulator. 

The V_HSY_ decreased from 1.46 ± 0.58 to 0.17 ± 0.03 V after SCG (Figure 8g,h). Overall, SCG also resulted in less variation in the extracted parameters, indicating better film uniformity. However, even after SCG, the hysteresis was still quite significant, especially in the off-state characteristics (Figure 7b). Additionally, there is still room to optimize the thermal treatment employed herein and further improvement can be achieved by employing additional strategies to suppress the formation of vacancies and ion migration such as the incorporation of multi cation mixtures and Lewis base acid treatments [53,54,55]. Additionally, various surface treatments such as the insertion of SAMs [16] and the use of additives to passivate surface defects [24,25,26,27,28,29] can be applied to further mitigate the difference in the surface energy or roughness between the G.I. and the perovskite semiconductor to achieve smoother pinhole-free thin films. 

## 4. Conclusions

The effect of crystallization process speed on the morphology of MAPbI_3_ thin films processed from a solution in ambient air was investigated. The crystallization speed can be controlled by varying the number of annealing steps, temperature, and resting time between steps. The resting period allows solvent-controlled growth (SCG) in which crystallization progresses slowly via an intermediate phase during which solvents slowly evaporate away from the films. SCG decreases the total number of defects and grain boundaries in MAPbI_3_—thereby significantly reducing hysteresis in MAPbI_3_ TFTs. 

To achieve SCG, a two-step annealing process is required. It is recommended to do the first step at approximately 70 °C followed by resting for at least one hour before the second step at approximately 120 °C. Adding steps between the 70 and 120 °C annealing or increasing the resting time does not significantly yield better results. Annealing at temperatures larger than 120 °C degrades the films due to the evaporation of MAI whereas annealing at temperatures lower than 70 °C is not sufficient for crystallization. A one-step annealing at 120 °C results in rapid crystallization from numerous crystal seeds, which leads to smaller grain size and voids in the film. This work demonstrates a simple strategy to obtain hysteresis-free perovskite devices in ambient air and thus opens up the scope of using organic–inorganic hybrid perovskites for applications beyond optoelectronic devices.

## Data Availability

Data sharing is not applicable to this article.
